# Bioremediating Oil Spills in Nutrient Poor Ocean Waters Using Fertilized Clay Mineral Flakes: Some Experimental Constraints

**DOI:** 10.1155/2013/704806

**Published:** 2013-06-23

**Authors:** Laurence N. Warr, André Friese, Florian Schwarz, Frieder Schauer, Ralph J. Portier, Laura M. Basirico, Gregory M. Olson

**Affiliations:** ^1^Institute for Geography and Geology, Ernst-Moritz-Arndt University, F.L. Jahn Strasse 17a, 17489 Greifswald, Germany; ^2^Institute for Microbiology, Ernst-Moritz-Arndt University, F.L. Jahn Strasse 15, 17489 Greifswald, Germany; ^3^School of the Coast & Environment, Louisiana State University, 1165 EC&E Building, Baton Rouge, LA 70803, USA

## Abstract

Much oil spill research has focused on fertilizing hydrocarbon oxidising bacteria, but a primary limitation is the rapid dilution of additives in open waters. A new technique is presented for bioremediation by adding nutrient amendments to the oil spill using thin filmed minerals comprised largely of Fullers Earth clay. Together with adsorbed N and P fertilizers, filming additives, and organoclay, clay flakes can be engineered to float on seawater, attach to the oil, and slowly release contained nutrients. Our laboratory experiments of microbial activity on weathered source oil from the Deepwater Horizon spill in the Gulf of Mexico show fertilized clay treatment significantly enhanced bacterial respiration and consumption of alkanes compared to untreated oil-in-water conditions and reacted faster than straight fertilization. Whereas a major portion (up to 98%) of the alkane content was removed during the 1 month period of experimentation by fertilized clay flake interaction; the reduced concentration of polyaromatic hydrocarbons was not significantly different from the non-clay bearing samples. Such clay flake treatment could offer a way to more effectively apply the fertilizer to the spill in open nutrient poor waters and thus significantly reduce the extent and duration of marine oil spills, but this method is not expected to impact hydrocarbon toxicity.

## 1. Introduction

**Figure 1:**
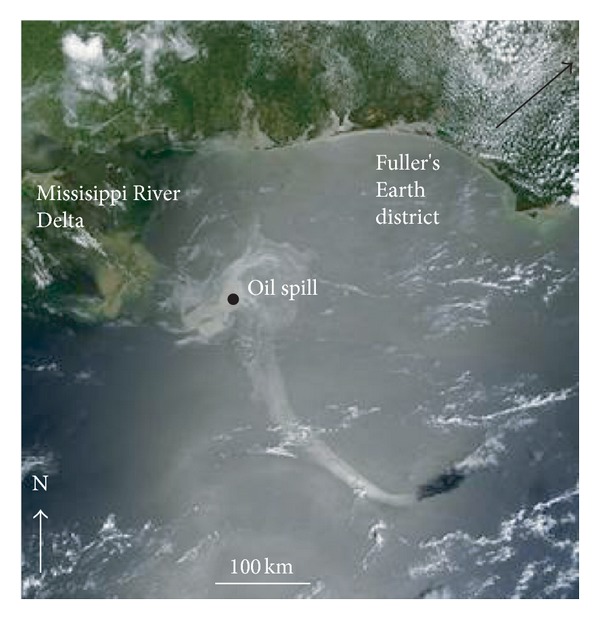
NASA's Terra satellite image of the Gulf of Mexico oil spill (light grey area) on May 17th, 2010, at 12:40 PM. The point marks the site of the Deepwater Horizon drilling platform that sank on April 20th, 2010 (source http://www.nasa.gov/home/index.html).

It is now estimated that 780 million litres of oil entered the Gulf of Mexico waters in the months following the sinking of the Deepwater Horizon platform on April 22, 2010 [1], affecting an area of over 75 000 km^2^ (Figure 1). After 7 months of clean up activity, it was considered that approximately 41% of the oil had evaporated, dissolved, or dispersed by natural means, 33% was captured, chemically dispersed, burned, or skimmed during remediation efforts, and 26% remained as a potential hazard [1]. British Petroleum's (BP) principle remediation effort used up to 3 000 000 litres of the dispersant Corexit (EC9500A and EC9527A), much of which was injected at 1500 m depth, to disperse the spill and reduce the amount reaching the surface [2]. Application of dispersants is controversial and in some environments has been shown to inhibit the activity of hydrocarbon-degrading bacteria [3], the primary mechanism by which oil eventually leaves the ecosystem.

In the case of both natural and anthropogenic oil spills, the bacteria necessary to metabolize hydrocarbons are likely present in the surface waters [4–8]. However, in the presence of large amounts of oil the C : N ratio is significantly enhanced and as a result the rate of degradation is diminished by limited growth of bacteria [9–11]. The application of P and N based fertilizer is therefore considered the most realistic approach to biostimulate the breakdown of the oil [2, 6, 9]. Such methods were extensively tested in the Exxon Valdez spill, with mixed success [2, 5, 12]. In the case of the Gulf of Mexico waters, fertilization was applied only locally in marshes where dispersion is not considered to be a significant problem [13]. The offshore waters of the Gulf of Mexico are oligotrophic in nature, and studies of bacterial activity in and around the spill of the Deepwater Horizon showed enhanced microbial respiration but signs of phosphate stress, indicating this nutrient was a factor limiting the rate of oil biodegradation [14]. The IXTOC I spill in the Bay of Campeche in 1979 spilled even more oil into these oligotrophic waters for more than a year. Oil covered almost 60% of the surface waters of the Gulf, and natural processes were the only option for oil mitigation/remediation. Thus, there is a general need to develop remediation strategies that overcome the limitations of poor nutrient supply. However, before fertilization can be successfully applied in open waters, a method is required for fixing the supplements to the oil so that bacteria can locally grow in abundance over sustainable time periods [6]. Some methods for developing oil dispersants containing oleophilic nutrients have been previous developed [15]. In this laboratory study, we investigate how treating a spill with thin-filmed fertilized Fullers Earth, a natural mineral material, available from neighbouring states, could improve the method of applying bacterial fertilizers to spills in open sea conditions. 

## 2. Materials 

### 2.1. Clay Minerals

Fullers Earth is sedimentary clay comprised typically of a mixture of palygorskite and Ca-montmorillonite. It was first used by the Romans as a detergent and found widespread application as a cleaning agent for wool during medieval times in a process known as fulling [16]. Today, North America provides over 70% of Fullers Earth for global industrial usage and is currently mined in south Georgia and the Florida panhandle (Figure 1). The Fullers Earth sample used was PF1-1 (palygorskite) from Gadsden Country, Florida, obtained from the Clay Minerals Society source clay collection (http://www.clays.org/SOURCE%20CLAYS/SCavailable.html). The main mineral constituents are 79% palygorskite, 11% Ca-montmorillonite, 10% quartz and feldspar, and 1% of accessory minerals, with a clay-sized fraction of high specific surface area (136 m^2 ^g^−1^) and a low cation exchange capacity (20 meq 100 g^−1^) [17]. The clays composition is composed of 60.90% SiO_2_, 10.40% Al_2_O_3_, 10.20% MgO, 2.98% Fe_2_O_3_, 1.98% CaO, 0.80% K_2_O, 0.49% TiO_2_, 0.80% P_2_O_5_, and 0.06% Na_2_O, with only traces of organic carbon (0.11% CO_2_ release) and oxidisable cations (0.40% FeO) present [18]. The main mineral component, palygorskite, with its fibrous and porous structure of tetrahedral layers joined by longitudinal side chains, has a good sorption capacity for organic molecules and is well known for its oil retention properties [19]. This palygorskite clay is also of interest due to its higher concentration of phosphorus (P_2_O_5_ 0.8%) compared to other types of reference clays [20]. We selected this clay for detailed testing as it simulated the most bacterial activity in our preliminary oil bioremediation experiments and was best suited for preparing thin mineral films that initially float in seawater. Other regionally available clays, namely, kaolin and Ca-bentonites available from Georgia and Texas, proved less successful.

### 2.2. Oil and Gulf of Mexico Water

The weathered source oil sample used was obtained on May 11, 2010 from the ruptured wellhead site (28° 44.20′N, 88° 23.23′W, Block 252, Mississippi Canyon, Gulf of Mexico). The composition of the oil was determined by gas chromatography/mass spectrometry analyses (GC/MS) and has an aliphatic to polyaromatic hydrocarbon (PAH) ratio of 29 : 1 (Table 3). Initial screening of the sample for viable biomass indicated that *Alteromonas*, *Marinobacter*, *Pseudomonas,* and *Vibrio* sp. were dominant within the microflora [21]. Similar profiles were seen in earlier studies of oil polluted coastal bays in pre-Katrina coastal Louisiana [22, 23]. Thus, the weathered source oil assemblages are more comparable to beach contaminated samples containing *Alcanivorax*, *Marinobacter*, *Pseudomonas, *and *Acinetobacter* [24] than the oil-degrading bacteria in the deep-sea plume [25]. For the experiments, a diluted Gulf coast water sample was collected from Galveston Bay (29°19′29.62′′N, 94°34′23.86′′W) in August 2010, with a salinity of 22 PSU. 

## 3. Experimental Procedure and Analytical Methods

### 3.1. Experimental Procedure

**Figure 2:**
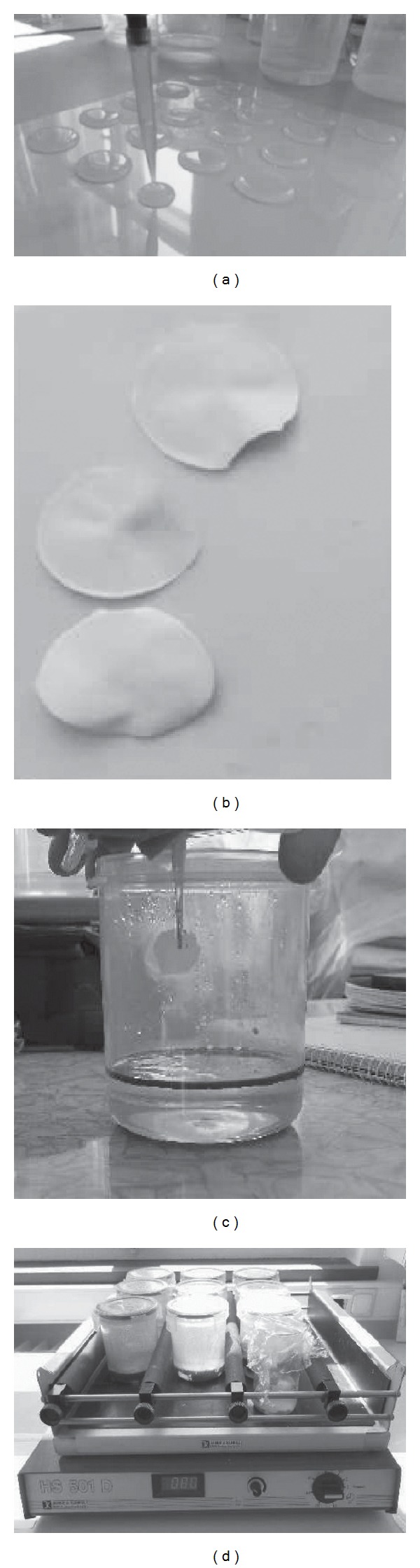
(a) Pipetting of clay-in-suspension (2 mL) drops onto plastic foil. (b) Removal of clay flakes after drying. (c) Placement of clay flakes onto the surface of the oil layer. (d) Continuous shaking of the sample containers on a shaking table during the 1 month duration of the experiment (100 motions per minute).

The clay flakes were prepared by first adding the filming agent, 0.25 g of sodium carboxymethyl cellulose (CMC), to 200 mL of distilled water and stirring until a dispersed solution was formed. Then, 2.5 g of PF1-1 and 0.25 g of the organoclay (Tixogel VP from Rockwood additives) were added to the solution as mineral powder, along with 10 mL of fertilizing solution, and stirred overnight. The fertilizing solution used was mixed according to Vyas and Dave [26], with optimum concentrations of N (1%), P (0.5%), and K (0.01%) applied as urea, phosphorus, and potash fertilizers. The homogenous clay-in-suspension was then pipetted onto plastic foil as 2 mL drops and allowed to dry (Figure 2(a)). The circular clay flakes then peeled off the substrate by bending the plastic (Figure 2(b)). The thin filmed clay used was 3 cm in diameter and 100–200 *μ*m in thickness and by mass consisted of 71.8% Fullers Earth, 7.1% CMC, 7.1% organoclay, and 14% N, P, and K fertilizer.

Laboratory simulations of the Gulf of Mexico were conducted using plastic beakers with the Gulf coast water (100 g), weathered Deepwater Horizon oil (5 g), and mineral flakes (0.5 g), giving a weight ratio of 100 : 5 : 0.5. Such a mixture is considered appropriate for investigating concentrated, thick oil spills, where nutrients will be limited, but is not representative of dispersed systems. The clay flakes were placed on the surface of the oil before gentle agitation using a table shaker (100 motions per minute) at an average temperature of 26°C (Figures 2(c) and 2(d)). To avoid excessive evaporation, the beakers were closed with caps and flushed twice weekly with pure O_2_ gas. Three experimental mixtures were prepared, the ingredients of which are listed in Table 1. Sample (1) contained water and oil, sample (2) contained water, oil, and fertilizer, and sample (3) contained water, oil, and the Fullers Earth clay flakes fertilized to the same concentration as in sample (2). 

### 3.2. Analytical Methods

To quantify bacterial respiration in the three experimental setups following a 1 month period of agitation, O_2_ consumption was measured in 7 mL of sampled water over a 100 hour period using an Hach HQ portable meter and a luminescent dissolved oxygen probe. The samples were taken from the same water depth within the beaker between the oil film on the surface and any mud at the base of the container. Some degree of heterogeneity in the O_2_ content of the waters is likely but repeat testing of the procedure did produce reproducible results. Prior to sampling of the water, the gas tight beakers were first flushed with pure O_2_ gas and then replaced on the shaker table for a period of 24 hours to allow the water to acquire dissolved O_2_. The rate of O_2_-depletion was then measured by placing the probe in the sampled oxygenated water within an open test tube placed in an slightly underpressured anaerobic box that kept the system air tight. Due to the small amount of water surrounding the probe, it was not necessary to stir the solution during measurement. Artifacts of the setup were excluded using an untreated Gulf coast water sample that during 100 hours of O_2_ measurement did not drop beneath 5.73 mL/L. O_2_ consumption by direct oxidation of the small quantities of mineral matter or oil present in the sampled waters was considered to be minor.

**Figure 3:**
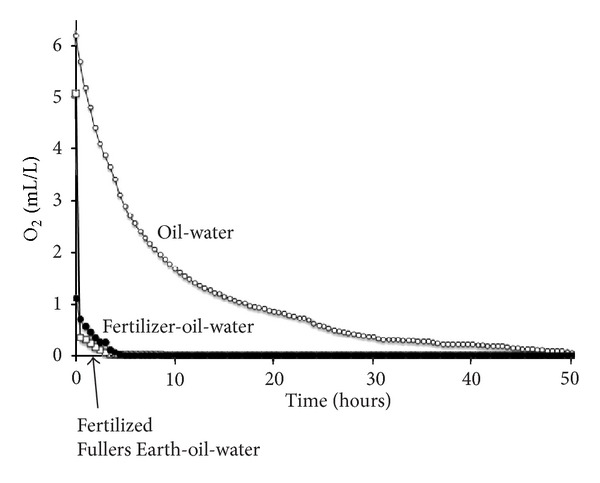
O_2_ depletion curves (mL/L) measured in 7 mL of Gulf coast water taken from the experimental beakers after 1 month of reaction time. Prior to sampling, the beakers were flushed with pure O_2_ gas and left for 24 hours to equilibrate.

The starting O_2_ concentration of the sampled waters was strongly influenced by the rate of diffusion between air and water within the beakers following the last O_2_ flush and varied between 1.11 to 6.22 mL/L (Table 1). As the presence of oil at the air-water interface limited the diffusion of O_2_ into the water, this process could not be well regulated. The moderately oxygenated conditions of samples 1 and 3 (starting concentrations of 5.09 and 6.22 mL/L) represented experiments containing local oil clumps or well dispersed oil droplets, whereas sample 2 (starting concentration of 1.11 mL/L) contained a thin dispersed oil film that inhibited oxygenation. Despite the initial differences in the starting concentrations, the O_2_ consumption curves approximate to exponential decay [27], and the time taken to consume half the O_2_ (Table 1) can be used to provide a general estimate of the overall rate of consumption caused by bacterial respiration (Figure 3). However, in the case of the lower O_2_ starting concentration of sample 2, the half concentration value is likely to underestimate the true rate of decrease rather than overestimate it due to the tendency of the O_2_ depletion to slow down significantly at very low concentrations.

Direct observations and microchemical analyses of the clay, bacteria, and oil components were made at the micron-to-nanometre scale on selected samples by transmission electron microscopy (TEM) combined with energy dispersive spectroscopy (EDS). Following dilution to reduce salt concentration, dispersed and diluted suspensions of the samples were sedimented onto carbon films that were stretched over a Cu-grid. This investigation used a JEOL JEM 1210 equipped with an OXFORD LINK EDX microanalyser, housed at the Geology Department of the University of Greifswald. The quantitative analysis was undertaken using the INCA software of Oxford instruments with internal calibration using pure Cu. Compositional analyses were made using the EDS mapping mode.

After the 1 month period of experimentation, the air tight beakers were refrigerated. Prior to hydrocarbon analysis, all moisture was first removed by lyophilisation. Extraction of alkanes and PAHs from the original weathered oil sample and experimentally treated oil samples was made by ultrasonic and solvent treatment for medium-high oil concentrations following the EPA method 3550c of the United States Environmental Protection Agency [28]. The concentration of 71 key constituents (Table 2) of the oil was determined by GC/MS analysis at the LSU Aquatic/Industrial Toxicology Laboratories, Baton Rouge, Louisiana, USA, following the EPA method 8270 series [29]. Based on these analyses, total alkane and total PAH concentrations were calculated (Table 3) together with total benzo(a)pyrene carcinogenic equivalents (BaP-TEQ) and total benzo(a)pyrene mutagenic equivalents (BaP-MEQ) [30, 31]. 

## 4. Results and Discussion

Following the 1 month of continuous agitation at an average temperature of 26°C, all of the sampled oil-treated waters showed rapid depletion of O_2_ that can be attributed to respiration by hydrocarbon-degrading bacteria (Figure 3). Other sources of O_2_ consumption such as the oxidation of cations (e.g., Fe^2+^) or organics in the clay flakes, or non-bacterial oxidation of hydrocarbons, were minimal due to the very low concentrations of reactive substances in the sampled waters. 

Bacterial respiration in the oil containing sample (1) was observed by rapid O_2_ consumption with 14 hours to half its concentration and total depletion to 0 mL/L by 50 hours. This compares to the sea-water control without oil that did not drop beneath 5.73 mL/L within the same time period. This sample was taken from the beaker which consisted of clear water with relatively unchanged floating clumps of weathered oil (Table 1). A major increase in the consumption of O_2_ was seen by adding the bioremediating fertilizer (2), whereby half the O_2_ was used in 1 hour and completely depleted within 5 hours. However, the addition of the fertilized thin filmed clay (3) led to the highest O_2_ consumption rate whereby half of the O_2_ was used up within 15 minutes, and total depletion was observed after 3 hours. 

**Figure 4:**
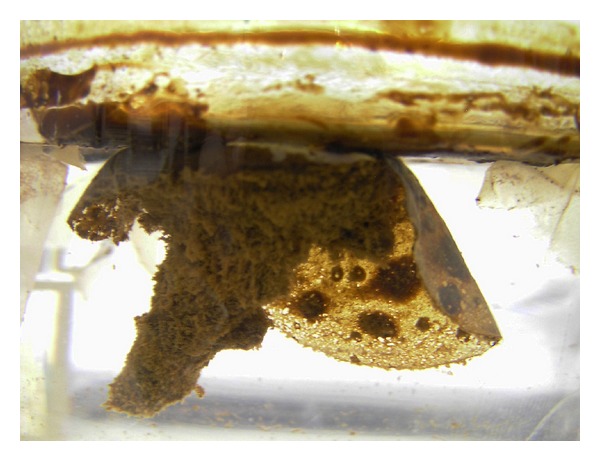
Photograph of a floating fertilized clay flake within Deepwater Horizon oil and Gulf coast water taken after 3 weeks of agitation. The clay flake is 3 cm in diameter. The circular dark patches on the base of flakes are clumps of oil. The brown furry mass attached to the flake consists of biofilm-clay-oil flocculates that slowly sink to form an organic-rich mud on the bottom of the beaker.

Compared to nontreated oil, the addition of fertilizer (sample 2) and fertilized clay (sample 3) increased O_2_ consumption rates by ×4.4 and ×17 times, respectively, despite equivalent concentrations of fertilizer added (Table 1). These experimental results indicate that the additional presence of the mineral flakes was responsible for an apparent 4 times increase in the rate of O_2_ consumption, a difference considered too large to be explained by inaccuracies in rate estimates. As no algal material was observed in the samples by electron microscopy, the measured O_2_ depletion curves are taken to reflect the respiration of hydrocarbon oxidizing bacteria present in the oil after 1 month of bioremediation activity and such respiration can be considered to be linearly related to the bacterial growth rate [32]. Both sample beakers treated with fertilizer show visual reduction in the amount of oil remaining. The straight fertilized sample consisted of a brown turbid water with oil coatings, whereas the fertilized clay treated sample contained the least visible oil with brown turbid water and dispersed oil droplets. During the experimental period, a large quantity of biofilm was also observed to form on the lower surfaces of the clay flakes (Figure 4), as well as on the beaker walls. Large amounts of biofilm production were particularly evident in all fertilized oil samples.

**Figure 5:**
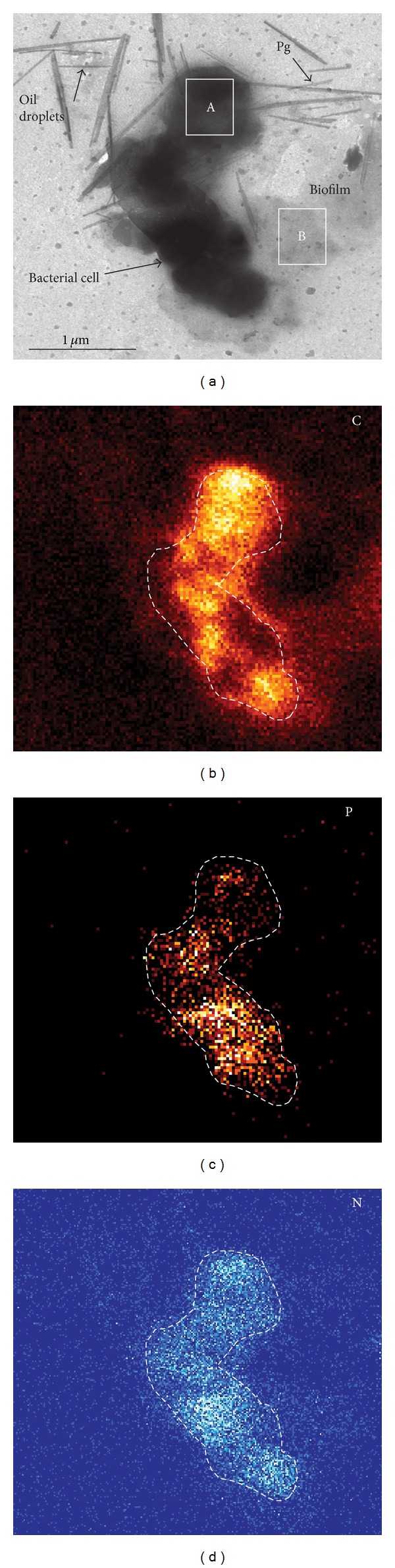
Transmission electron microscope image and selective elemental maps for C, P, and N of the clay-treated oil showing relationships between bacteria, oil, and palygorskite (Pg) clay mineral particles. Weight (%) element composition of analysis A (bacterial cell) in decreasing abundance is C 26%, O 28%, Na 18%, K 10%, N 5%, Si 4%, S 3%, Mg 2%, Ca 1%, Cl, Al, P, and Fe each >1%. Weight (%) element composition of analysis B (biofilm) in decreasing abundance is C 28% O 16%, Na 8%, K 17%, Cl 14%, N 7%, S 5%, Si 2%, Mg 1%, Ca 1%, Al, and P each <1%.

Transmission electron microscopy (TEM) investigation of the dried fine-grained solids of three experiments containers (Table 1) revealed abundant bacterial cells present in all samples. The highest concentration of cells and biofilm were observed in the dispersed oily-flocculates taken from the clay treated samples, together with dispersed oil droplets and palygorskite clay mineral fibres (Figure 5). The bacteria cells were recognizable by their size and shape, the occurrence of a membrane, the nature of internal structures (e.g., nuclei and phosphorus rich globules), and compositional characteristics [33]. The bacterial cells in the clay-treated oil sample range in size between 0.5 and 2.3 *μ*m and show frequent signs of cell division. The cells were also encrusted in salts (NaCl, KCl, and sulphates) formed after desiccation of the Gulf coast water. The distribution of organic C (weight %) confirms the position of bacterial cells which are typically surrounded by a mass of biofilm and submicron dispersed droplets of oil (average size of 0.24 *μ*m). These areas also show enrichment in P and N derived from the fertilizer, as well as notable enrichment in K (17 weight % in the biofilm), indicating that nutrients were not limiting factors for bacterial activity in this experiment (Figure 5). Some P may also be derived from the Fullers Earth (P_2_O_5_ = 0.9%), which is expected to release its nutrients more slowly than the fertilizing agent. The high concentration of N (7%) present in the biofilm may also be partially derived from the organoclay component, which consists of an quaternary ammonium compound of smectite.

The overall impact of bacterial degradation on the weathered Deepwater Horizon source oil is revealed in the GC/MS hydrocarbon compositions determined at the end of the 1 month experimental period (Table 3). The oil-in-water beaker (1) contained significantly less alkane (1124 mg/kg) and less PAH (122 mg/kg) than the original source oil sample (0), representing a reduction of 82.5% and 43.5%, respectively. The large decrease in alkane content reflects the bacteria's preference for consuming straight chained hydrocarbons. Addition of fertilizer significantly enhanced the reduction of both alkane and PAH content (to 267 mg/kg and 101 mg/kg), representing a 95.8% and 53.2% decrease. However, the highest degree of alkane degradation was achieved by the fertilized clay treatment (130 mg/kg remaining), representing a reduction of 98%. However, the total PAH content on this sample (3) was not the lowest (126 mg/kg: a 41.7% reduction). The overall variation in PAH content between 101–126 mg/kg measured for samples 1, 2, and 3, probably reflects sampling heterogeneities. Such heterogeneities also appear to influence the BaP-TEQ amd BaP-MEQ indicators, with experimental values ranging between 1.1 and 1.9 (mg/kg). Compared to the original source oil, these values represent a reduction in toxic and mutagenic components by 25%–45%. Despite the overall reduction in PAHs and toxicity/mutagenic indicators compared to the original weathered source oil (Table 3), treatment by straight fertilization (sample 2) or fertilized clay flakes (sample 3) did not lead to significantly different PAH concentrations than measured in the Gulf coast water-oil experiment lacking additives (sample 1).

We propose that these laboratory simulations hold important implications for more effective bioremediation of oil spills in open marine waters. The presence of fine mineral particles in oil spill is well documented and is known to improve both oil dispersion and bacterial activity [33–36]. However, here we propose that specially prepared thin filmed clay, loaded with fertilizing agents, can successfully target the nutrients to the oil and thus sustain long term degradation by bacteria. Our experimental results indicate that limitations in phosphorus and/or nitrogen that prevented more advance biodegradation of the Deepwater Horizon oil could be over come by targeted clay treatment. 

Fullers Earth is proposed as the most suited for this purpose whereby the problem of sinking is over come by preparation as dried thin mineral films that initially float on the surface tension of the water and subsequently have a prolonged resident time in the upper part of the water column. The clay films may remain buoyant as oil is adsorbed to organoclay particles surfaces or entrapped between palygorskite fibres. An additional advantage of this type of thin filmed clay is that it can be applied without releasing large quantities of undesired dust [37] that is produced when applying mineral powders. Based on experimental observation (Figure 4), the clay that interacts with the oil forms biofilm-clay-dispersed oil flocculates that disperse and sink to form an organic rich mud, whereas filmed clay that does not attach to floating oil will slowly disperse and sediment. The formation of such clay-organic flocculates is considered favourable for the continued breakdown of oil droplets due to the increase in oil-water interfacial area, which further enhances access to the oil by bacteria for degradation [38]. The negatively charged clay mineral particles may also play a role in adsorbing any toxic metals or oxidized organic compounds that can be detrimental to bacterial growth [33].

Although field testing is required to verify our experiments, the results do define a new approach to bioremediation in open oligotrophic waters that could enhance bacterial degradation of oil and hence reduce the environmental and financial impact of this form of marine pollution. As the basic ingredients of thin filmed clay are cheap, with an average 2012 price for US Fullers Earth being $110 per tonne [39], this method may cost less than applying expensive chemical dispersants and could reduce the amount of fertilizer needed by limiting nutrient dispersion in open waters. Importantly, such clays are available in industrially large quantities, are located in regional proximity to the Gulf of Mexico, and can be easily prepared by simple wetting, mixing, and drying procedures. Preparation could be undertaken at the clay pit, or alternatively in closer proximity to the site of spillage. Whatever the best logistics, the potential cheapness of fertilized clay could represent an attractive alternative to achieving efficient and cost effective cleanup for lengthy and voluminous oil spills situated in the open expanses of our planet's oceanic environment.

## 5. Conclusions

1. Mineral flakes, composed of thin clay mineral films that contain a mixture of Fullers Earth, organoclay, sodium carboxymethyl cellulose, and urea, phos, and potash fertilizers can be made to float on water and attach to oil spills in marine waters. This is presented as a method of concentrating fertilizer in the oil and to overcome the problem of nutrient dispersion in open waters.
2. Laboratory experiments indicate that the application of fertilized clay mineral flakes can significantly enhance the rate of bacterial respiration compared to both untreated oil-water conditions and straight fertilization without clay. After a 1 month reaction period, such treatment removed 98% of the total alkane concentration compared to 82.5% in the sample lacking additives. Despite the large reduction in alkane content, the total concentration of PAHs and toxicity and mutagenic indicators were not reduced further by such treatment.
3. A large portion of the oil was converted to bacterial biofilm, which sedimented in the water coloumn as clay-organic flocculates. Small quantities of oil in the form of micron-sized droplets remained in the flocculates.

## Figures and Tables

**Table 1:** O_2_ concentrations (ml/L) in solution (shown in Figure 3) measured over a period of 100 hours. The samples (1–3) were extracted and measured 24 hours after O_2_ flushing of the gas-tight batch experiments.

	(1)	(2)	(3)
	Gulf coast water	Gulf coast water	Gulf coast water
	Oil	Oil	Oil
		Fertilizer	Fertilized clay
Start O_2_ (ml/L)	6.22	1.11	5.09
Time to half O_2 _	4.4 hr	1 hr	<15 min
Time to 0.01 ml/L	86.5 hr	9 hr	5 hr
Rate enhancement	Control	×4.4	>17.6
Observations (after 2 months)	Clear water with floating clumps of oil	Brown turbid water with thick oil coatings on wall of container	Brown turbid water with oil droplets

**Table 2:** 71 alkane and PAH compounds quantified using a modified GC/MS method.

Internal standard	n-Alkanes	n-Alkanes	PAHs
Napthalene-d8	nC-10 Decane	nC-22 Docosane	Naphthalene, C1–C4*
Acenaphthene-d10	nC-11 Undecane	nC-23 Tricosane	Fluorene, C1–C3*
Chrysene-d12	nC-12 Dodecane	nC-24 Tetracosane	Dibenzothiophene, C1–C3*
Perylene-d12	nC-13 Tridecane	nC-25 Pentacosane	Phenanthrene, C1–C4*
Surrogate Standard	nC-14 Tetradecane	nC-26 Hexacosane	Anthracene
Phenanthrene-d10	nC-15 Pentadecane	nC-27 Heptacosane	Fluoranthene
Androstane	nC-16 Hexadecane	nC-28 Octacosane	Pyrene, C1–C4*
	nC-17 Heptadecane	nC-29 Nonacosane	NBT, C1–C3*
	Pristane	nC-30 Triacontane	Benzo (a) Anthracene
	nC-18 Octadecane	nC-31 Hentriacontane	Chrysene, C1–C4*
	Phytane	nC-32 Dotriacontane	Benzo (b) Fluoranthene
	nC-19 Nonadecane	nC-33 Tritriacontane	Benzo (k) Fluoranthene
	nC-20 Eicosane	nC-34 Tetratriacontane	Benzo (e) Pyrene
	nC-21 Heneicosane	nC-35 Pentatriacontane	Benzo (a) Pyrene
			Perylene
			Indeno (1,2,3-cd) Pyrene
			Dibenzo (a,h) anthracene
			Benzo (g,h,i) perylene

*Indicates parent compound and associated homologues.

**Table 3:** Summary of the hydrocarbon analyses by GC/MS showing the totals (mg/kg) of alkane and PAH contents, as well as totals (mg/kg) of BaP-TEQ and MEQ-TEQ indicators.

	(0)	(1)	(2)	(3)
	Original source	Gulf coast water	Gulf coast water	Gulf coast water
	Oil*	Oil	Oil + fertilizer	Oil + fertilized clay
Total alkanes (mg/kg)	6422.0	1123.7	267.4	130.0
Total PAH (mg/kg)	216.0	122.0	101.5	126.5
Total BaP-TEQ (mg/kg)	2.8	1.3	1.9	1.8
Total BaP-MEQ (mg/kg)	2.0	1.2	1.1	1.5

*Average of 2 sample analyses.
